# The Creation of Shared Mental Models in Simulation Training Enhances Quality of Resuscitation: A Randomized Controlled Study

**DOI:** 10.1177/23821205251316749

**Published:** 2025-03-02

**Authors:** Christopher Friederich, Leonie Schulte-Unetrop, Denisa Cenaj, Leonie Fée Kröger, Josephine Küllmei, Christian Zöllner, Parisa Moll-Khosrawi

**Affiliations:** 1 Department of Anaesthesiology, 37734 University Medical Center Hamburg-Eppendorf, Hamburg, Germany

**Keywords:** simulation-based medical education, cardiopulmonary resuscitation, non-technical skills, mental modeling, motivation

## Abstract

**OBJECTIVES:**

This study aimed to enhance the quality of Advanced Cardiac Life Support (ACLS) training, with quality defined as the combination of technical skills (TS) and non-technical skills (NTS), by addressing the gap in effective methods for developing NTS through simulation-based medical education (SBME). Specifically, it sought to develop and evaluate a strategy for establishing shared mental models (SMM) and fostering trust among team members during undergraduate emergency training.

**METHODS:**

This study was conducted during mandatory ACLS undergraduate simulation training sessions. The control group participated in traditional, teacher-led classes and debriefings, while the intervention group received training incorporating SMM as the intervention. The study evaluated the quality of cardiopulmonary resuscitation as the primary outcome, encompassing both TS and NTS. Additionally, changes in undergraduate situational motivation, assessed within the framework of self-determination theory, and subjective learning gains were analyzed.

**RESULTS:**

The control group demonstrated a significant improvement in TS (*P* = .030), while the intervention group did not (*P* = .078). Conversely, the intervention group showed a significant improvement in NTS (*P* = .01; 95% confidence interval [0.296, 2.17]), whereas the control group did not (*P* = .105). The motivational changes of both groups were comparable, reflecting high levels of autonomous motivation. Both groups also reported significant learning gains.

**CONCLUSION:**

This study demonstrates that SBME is highly effective for teaching TS. However, it is crucial to incorporate advanced instructional methods focusing on NTS. One promising approach is the development of SMM. Based on our results, hands-on practice remains essential and should not be restricted to theoretical or conceptual training. A balanced combination of advanced didactic techniques and practical application ensures that learners develop both, TS and NTS. SBME and the development of SMM equally address both the motivational and content dimensions of learning, enhancing student engagement while effectively conveying essential knowledge and skills.

## Introduction

High-quality cardiopulmonary resuscitation (CPR) enhances patient outcomes and reduces mortality after sudden cardiac arrest.^1,2,3–4^

The backbone of resuscitation efforts is to keep the “no-flow time” as low as possible and hence provide vital organs with oxygen.^4^ Interruption of chest compressions reduces myocardial blood flow (low cardiac perfusion pressures)^3^ as well as brain circulation and results in a decreased 24-h survival rate.^2^ The skill components of CPR contributing to the “no-flow time” are: chest compression rate, chest compression depth, chest recoil, chest compression fraction, and ventilation.^3^

To convey these technical skills (TS), simulation-based training has been identified as a suitable didactic method.^5,6–7^ However, even if resided outside the core curriculum of CPR for a long time, non-technical skills (NTS) have been described as just as important as TS regarding patient outcome.^5^ Therefore, the quality of CPR is also determined by the NTS of the response team.^8,9^

The term NTS refers to an individual set of cognitive, social, and personal abilities that complement TS and are crucial for patient safety.^10^ Deficiencies of NTS increase the chances of human error^11,12–13^ with up to 82% of medical mishaps depending on a lack of NTS.^14^

The latest resuscitation guidelines emphasize the importance of incorporating NTS training into Advanced Cardiac Life Support (ACLS) courses. However, the best approach to teaching them remains unclear.^15^ As a first step, it is essential to define NTS within an educational framework. In our previous work, we identified three key dimensions particularly relevant for undergraduate education in emergency medicine settings: 1. Planning tasks, prioritizing, and problem-solving; 2. Teamwork and leadership; and 3. Team orientation.^11^ To effectively realize these components of NTS, it is crucial that team members trust each other and develop shared mental models (SMM).^16^ Consequently, fostering SMM during simulated emergency training could serve as an effective didactic approach to enhance NTS. Mental models (MM) are information in a person's mind used to understand the external world.^17^ MM can be understood as an individual’s internal representation of real patterns, such as team tasks, responsibility, or task sharing.^17,18^

When individual MM symbiose to a common understanding of the team, SMM are generated. This process is fostered and strengthened by team training and is therefore well-suited for undergraduate education to enhance NTS.^19,20^

It has already been demonstrated that the SMM of team members enhance team effectiveness and hence, teamwork.^21,22^

Applying the concept of SMM during undergraduate resuscitation training could lead to additional positive outcomes, such as increased student motivation. The motivational aspect of learning is often overlooked, despite its repeatedly emphasized significant impact on the learning process.^23,24–25^ The leading motivational theory in the field of medical education is the “self-determination theory” (SDT), described by Deci and Ryan.^26^ SDT moves away from the dichotomous description of individual motivation and postulates, that every human being has the innate will to grow. This growth is determined by how much three basic psychological needs—autonomy, relatedness, and competence—are satisfied.^27^ Consequently, motivation is described as a continuum, with amotivation (having no motivation) as its one end and intrinsic motivation as its other.^28^ Every motivation has an underlying behavioral regulation and regulatory process, as well as a locus of causality.^27^ Two main types of behavioral regulation can be found in human beings to carry out an activity: Autonomous and controlled regulation. The most autonomous type of motivation is intrinsic motivation, which is usually present when an activity is carried out based on inherent satisfaction.^27^ When external sources (punishment or rewards) form the basis of activities, extrinsic motivation is foregrounded. SDT describes four types of extrinsic motivation, all with varying levels of autonomy:^29^ The least autonomous is externally regulated behavior, which results from external demands, possible rewards, or punishment.^30^ When an activity is less due to external factors and more autonomous than external regulation, introjected regulation is foregrounded.^30,31^ Introjected behavioral regulation is mostly at hand when it comes to avoid guilt and attain self-esteem and citing ego.^29,31,32–33^ Moving further toward autonomous motivation on the continuous motivational scale, identified regulation is more autonomous than introjected regulation. The individual accepts the action inwardly as important and therefore values the goal.^33,34^ Intrinsic and identified regulation are summarized as “autonomous self-regulation,” whereas extrinsic and introjected regulation are summarized as “controlled self-regulation.”

Amotivation is foregrounded when a person experiences a lack of competence or does not see the reason for a task and hence has no motivation.^29,35^

High levels of autonomous motivation in medical students are aspirational as autonomous motivation has been linked to several positive outcomes like better learning, better academic achievement, better well-being, perseverance, and enthusiasm. Reducing controlled regulation should also be focused on, as it has been associated with more negative outcomes in medical students.^24,36,37–38^

Although theoretically, applying the concept of SMM in undergraduate education appears to be a promising method, the most effective way to implement SMM to improve resuscitation skills and positively impact the motivational aspect of learning remains unclear.

Therefore, in this prospective randomized controlled study, we developed and assessed a concept for establishing SMM in simulated undergraduate ACLS training. The quality of CPR in the intervention group, which had a build-in unit to develop SMM, was compared to a control group which had conventional ACLS training. The quality of CPR was composed of NTS and TS (primary outcome), and the secondary outcome was the motivational change in undergraduates. The subjective learning gain of the undergraduates was the tertiary outcome. We hypothesized that the intervention of building SMM would have a positive effect on all outcome parameters.

## Methods

The reporting of this study conforms to the CONSORT statement.^39^ (Supplementary File 1).

### Study design and setting

This prospective randomized controlled simulation study with blinded participants was performed from April 2023 to July 2023 at the Department of Anaesthesiology, University Medical Center Hamburg-Eppendorf.

The undergraduate medical curriculum at the University of Hamburg is an integrated curriculum based on learning spirals.^40^

Accordingly, undergraduates partake in different emergency simulation trainings in almost every year of medical school that build upon each other. In the third year of medical school, Basic Life Support (BLS)—as well as ACLS training has already been passed by the students in the preceding semesters. The third training thus serves as both a refresher and an advanced learning opportunity: The undergraduates participate in two trainings, which are scheduled within 1 week (Tuesday and Thursday). Each training has a time span of 120 min. The trainings take place throughout the whole semester and the undergraduates are randomly allocated to smaller subgroups (cohorts) by the deanery in advance, to have a maximum of 18 students per training. The trainings have predefined learning objectives which are available on an online platform.

After a brief theoretical repetition of the ACLS algorithm, participants are divided into smaller groups of three to six to complete different simulation scenarios. The scenarios are standardized for each training and are similar regarding the requirements for NTS and TS. All simulated scenarios are cardiothoracic emergencies, with initially awake and responsive patients, that end in cardiac arrest, with the need to perform CPR based on the ACLS algorithm.^4^

For each scenario, smaller subgroups of undergraduates take on the roles of a physician and two paramedics. The remaining undergraduates take over an observational role.

The trainings are conducted with high-fidelity manikins (Resusci Anne QCPR manikin, Laerdal Medical AS, Stavanger, Norway) to simulate the scenarios as real as possible: The instructors have the possibility to activate and individually adjust vital signs shown on the monitor, depending on the quality of performed CPR and the medical measures taken. Undergraduates can train TS like CPR, defibrillation, endotracheal intubation, vital sign monitoring, and drug administration.

Each scenario is supervised by residents of the Department of Anaesthesiology, who are experienced in the practical and theoretical teaching of undergraduates and regularly trained in cardiac life support, as they are also clinically active, working with patients. After completing a scenario, structured feedback is provided by the supervising instructor. The debriefing is held in the conventional way and the role of the instructor during the debriefing is that of a teacher.

The training of the control group was carried out as specified in the curriculum. The training of the intervention group was modified by the intervention. The structure of both trainings, including the learning objectives and the scenarios were identical. The intervention took place at the end of the first training (Tuesday). Consequently, the Tuesday training sessions for both groups served as the baseline.

To prevent bias caused by technical application problems, we made sure that all instructors were sufficiently trained in advance regarding the use of the evaluation forms and high-fidelity manikins.

### Study procedure

**Figure 1.**
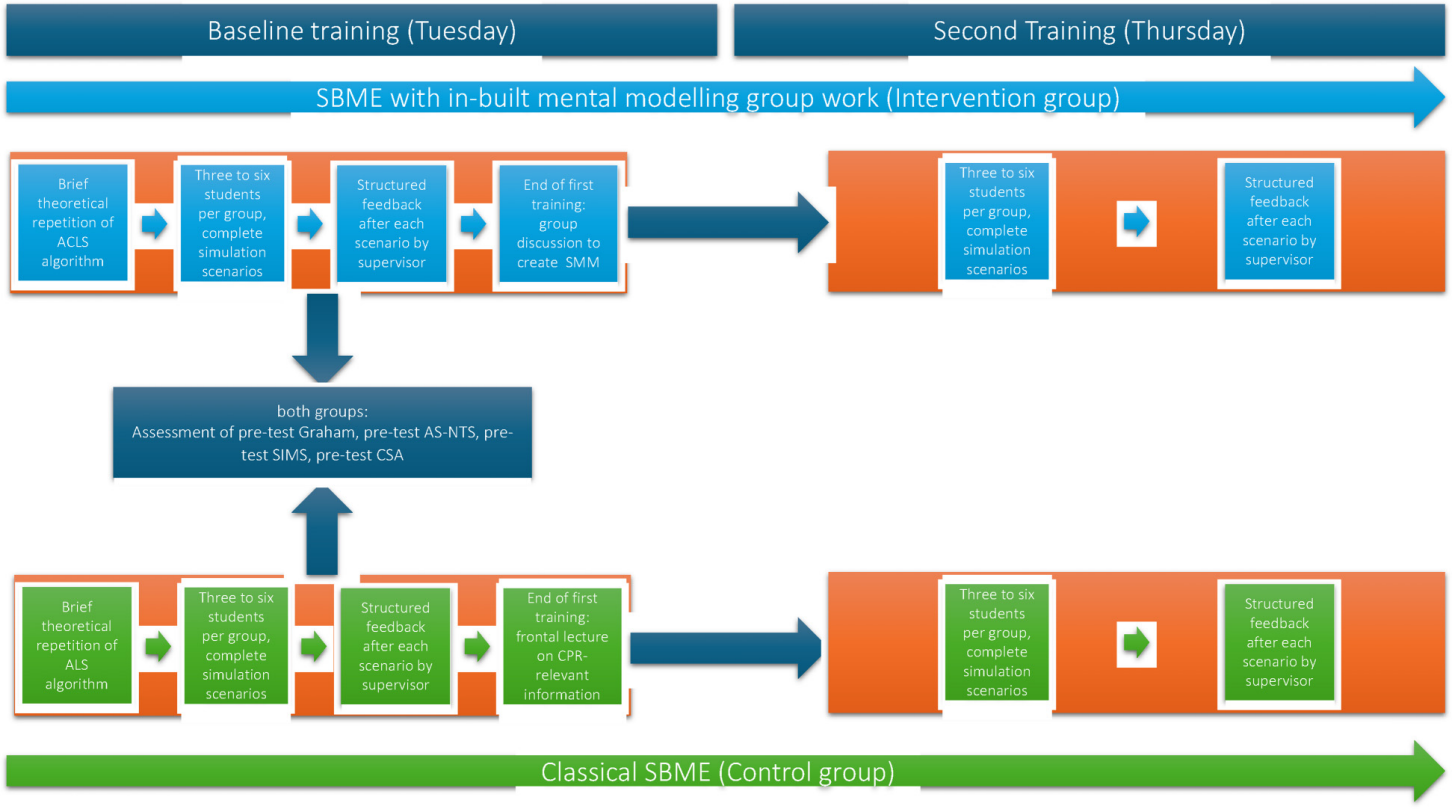
Study procedure.

*Note*. The top rectangles represent the training sessions for the intervention group (blue), while the lower rectangles represent the training sessions for the control group (green). The difference between the two baseline training of the intervention and control groups is emphasized by the dark blue rectangles. Abbreviations: ALS, advanced life support; SIMS, Situational Motivation Scale; CPR, cardiopulmonary resuscitation; AS-NTS, Anesthesiology Students' Non-Technical Skills; Graham, Graham scoring, adapted by Graham and Lewis;^41^ CSA, comparative self-assessment; SBME, simulation-based medical education.

The study procedure is depicted in Figure 1.

Before the study, the research group drafted a semi-structured guide for the group discussion (intervention) through several qualitative analysis steps. This guide primarily aimed to identify key outcome measures of CPR while considering important behavioral patterns.

### Intervention group and intervention

After the scenarios were completed (end of the first training), all the subgroups came together, and the intervention was conducted. A semi-structured group discussion with open questions was carried out with the undergraduates, aiming the goal to create SMM of all the team members.

During the group discussion, undergraduates were encouraged to identify key points independently to foster the creation of a thorough SMM regarding tasks, timing, and responsibility. The anticipated responses had been predetermined by the research team during the qualitative analysis phase. Consequently, the questions in the discussion guide served as prompts for the undergraduates. The discussion and its outcomes were effectively presented throughout the intervention using strategic visualization techniques.

Throughout the study period, the intervention was always carried out by the same individuals: One fellow undergraduate student (third year of medical school) and one resident of the Department of Anaesthesiology.

#### Group discussion

For the first question (“Name the three most important outcome relevant measures of CPR”) we assumed “low no-flow time,” “early defibrillation of shockable rhythms,” and “quality of CPR” as the most crucial points. The undergraduates were given further information on the individual measures and had the opportunity to clarify questions. The second question (“How can we achieve good CPR as a team?”) targeted the importance of using NTS to integrate effective communication, feedback loops, and correcting each other on a factual level into medical care to enhance CPR performance. The undergraduates were encouraged to identify both positive and negative factors influencing teamwork by themselves. To support the learning cycle and learning retention by reflection, the participants were asked to create a shared flip chart at the end of the class, which included the aspects they wanted to focus on as a team at the second training (Thursday) to enhance their performance.

### Control group

The control group essentially followed the same training structure but without the group discussion component. They received a brief lecture delivered by the instructors, focusing on the most important outcome-relevant resuscitation measures. These measures were summarized on PowerPoint slides and presented as a traditional lecture to all participants. Both TS and NTS were addressed and concisely summarized. The undergraduates were encouraged to brainstorm on each topic. For instance, one slide illustrated common pitfalls in CPR situations where communication and teamwork might fail, while another slide offered tips on reducing no-flow time.

The second training (Thursday) had the same structure for both groups and was composed of different simulation scenarios.

### Participants

Third-year medical students who attended mandatory ACLS training were eligible and enrolled in the study. We selected medical undergraduate students in their third year of studies, as they already were familiar with simulation-based emergency training, to prevent potential bias caused by being overwhelmed by the situation or emergencies. Participation in the study was voluntary. An e-mail with a detailed description of the study, as well as a declaration that they would not suffer any disadvantages if they did not participate, was sent to all eligible participants. Written informed consent was obtained in advance from each study participant. Each cohort was randomly assigned to the intervention group or control group by computer-generated random numbers.

### Data collection and assessment tools

The time points of assessments are already depicted in Figure 1 above.

#### Primary outcome: quality of CPR, TS, and NTS

TS and NTS were assessed for each simulated scenario on Tuesday (baseline) and Thursday (post-intervention/control).

To assess the quality of CPR (TS), the instructors filled in the “Graham-Score”, adapted from Graham and Lewis,^41^ after each scenario (one score per scenario/team). This assessment tool has originally been developed to assess the quality of BLS.^41^ In a pilot study, we have expanded and adapted the scoring for ACLS.

TS are rated on a total of 12 items and the given points are considered as penalty points. The points are given, according to predefined performance. Therefore, penalty points are awarded for incorrect performance of each ACLS component, based on the potential to compromise patient safety. The best possible ACLS performance is combined with zero penalty points, while the worst performance is with 200 penalty points.

NTS performance of the team leader (the student taking the role of the physician), was assessed using the German version of “Anaesthesiology Students’ Non-Technical Skills” (AS-NTS).^11^ A validated rating tool has been developed to assess NTS directly during simulation training. On AS-NTS, NTS skills are rated on three dimensions:

- “Task planning, prioritization, and problem-solving”
- “Teamwork and leadership”
- “Team orientation.”

For each dimension, NTS are rated separately on a five-point Likert scale (1 = “very good”; 5 = “very poor”). Examples of corresponding very good or poor performances in the respective of each dimension are provided on the AS-NTS.^11^

As a sub-analysis, the correlation between NTS and TS was computed.

#### Secondary outcome: situational motivation to participate in training

Situational motivation to participate in each training was assessed with the German Version of the “Situational Motivation Scale” (SIMS), which was developed based on the SDT of motivation.^26,42,43^ The reliability and validity of the SIMS, as well as the German translation, have been confirmed in many studies.^42,43^ The students filled out a paper-based SIMS questionnaire at the beginning of the first (pre-test) and at the beginning of the second (post-test) training.

The SIMS measures the underlying motivation to participate in a task or activity at a specific point of time (situational), on four subscales (intrinsic motivation, identified regulation, introjected regulation, external regulation, and amotivation) which contains 20 items. Each item has a seven-point Likert scale (1 = Does not correspond at all; 7 = Corresponds exactly).

Autonomous motivation is computed by adding and averaging intrinsic motivation and identified regulation provides autonomous motivation. Controlled regulation is computed by adding and averaging external regulation and introjected regulation.^42^

#### Tertiary outcome: subjective learning gain of the students

The tertiary outcome was the learning gain, which was assessed by a validated self-assessment tool, the comparative self-assessment (CSA)^44^ which assesses the learning gain and in the adapted for our study, that of ACLS.

This self-assessment tool contains 11 questions, for each question, a six-point Likert scale is given (1 = “mostly applies”; 6 = “does not apply”). The students filled out the CSA before the first and after the second training.

### Statistical analysis

Statistical analysis was performed using IBM SPSS Statistics Version 29.0.2.0.^19^

Descriptive statistics were used to calculate the mean values for each AS-NTS dimension, Graham score criterion, CSA elements, and for computing the subscale scores of the SIMS.

For each analysis, the two-stage *P*-value was determined at a significance level of .05.

The disparities between pre-training and post-training sessions were compared by conducting score subtractions: CSA gain (points) = CSA_pre_ – CSA_post_. Participants who rated themselves with the highest possible score (1 = “mostly applies”) in the pre-training self-assessment were excluded from the analysis, since no learning gain can be observed in relation to these points.

For the intracohort analysis, a paired *t*-test was calculated for all outcome parameters. The assumptions of the paired *t*-test were not violated by our data. There were no outliers and the differences between the pre- and post-test scores were normally distributed as assessed by the Shapiro–Wilk test. Homogeneity of error variances in each group, as assessed by Levenés test (*P* > .05) was given.

An unpaired *t*-test was conducted to analyze the differences between groups at baseline and post-test. Normal distribution of the data and equal variances between the groups was given.

## Results

### Participants

**Figure 2.**
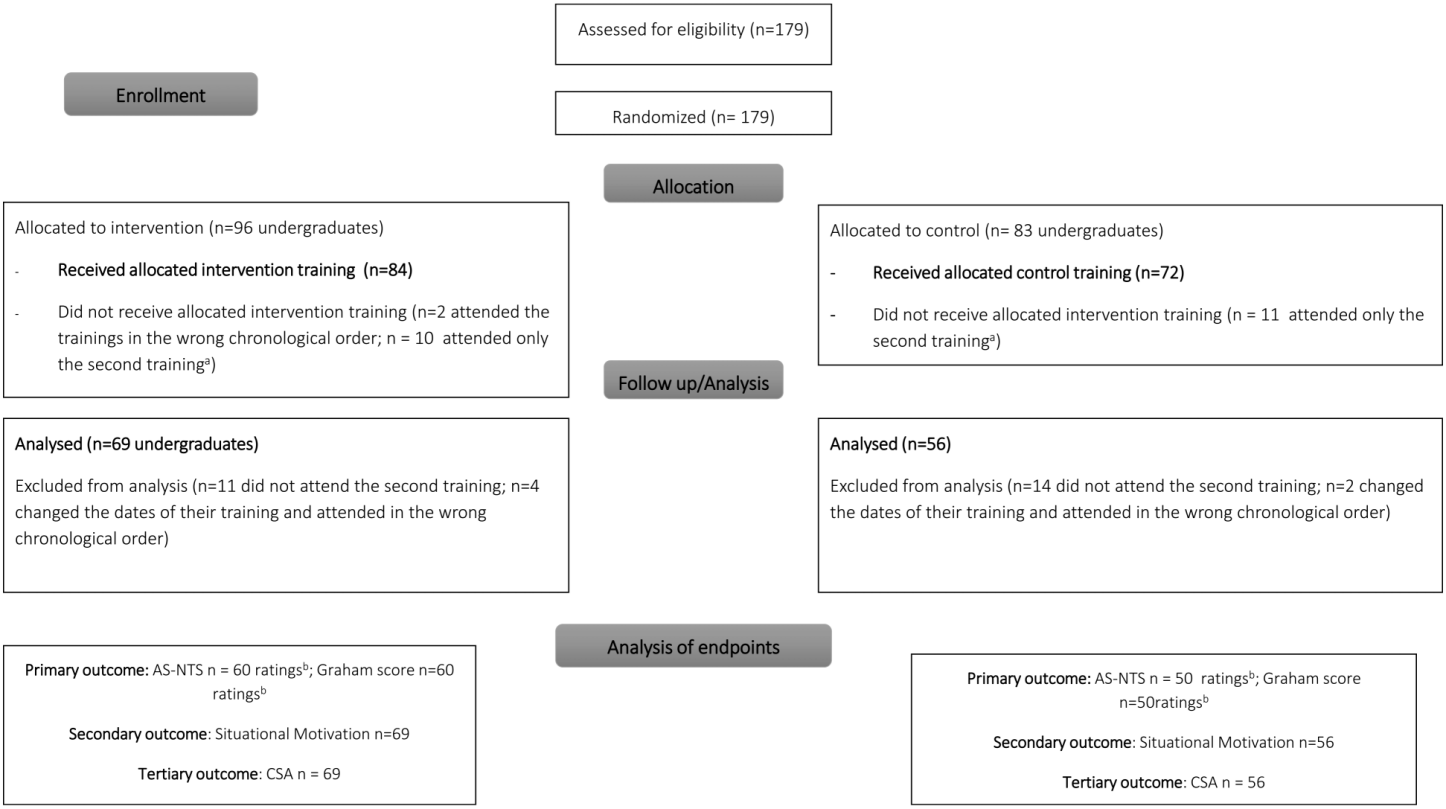
Participant flow of the study.

Abbreviations: AS-NTS, Anesthesiology Students' Non-Technical Skills; CSA, Comparative self-assessment.

^a^Cancelation of training due to public holidays and public transport strikes. ^b^Subgroups of students simulated the scenarios and the AS-NTS and the Graham score were assessed for each scenario, therefore the number of assessments is smaller than the total number of analyzed students.

A total of 179 students were enrolled in the study and 125 of them (*n* = 69 intervention-, *n* = 56 control group) were included in the final analysis. The inclusion criteria were the attendance of both trainings in the correct sequence, as the analysis was based on repeated measures. The dropouts were mainly because some trainings being canceled without substitution due to public holidays. Also, there was a public transport strike on one training date, which also led to lessons being canceled. Furthermore, some students changed their training date which led to an incorrect training sequence (Figure 2).

As shown in Table 1, the demographics of the randomized and analyzed students were not significantly different.

**Table 1.** Demographic data of the study participants included in the final analysis.

	INTERVENTION GROUP (*N* = 69)	CONTROL GROUP (*N* = 56)	*P*
*M*_age_ (range), years	22.4	22.9	>.05
Gender, *n* (%)			>.05
Female	36 (52.2)	29 (51.8)	>.05
Male	33 (47.8)	27 (48.2)	
Additional CPR and/or emergency training. (Despise the training of the curriculum) Prior medical knowledge or medical work experience (training as a paramedic nurse etc.), *n* (%)	2 (2.8)	1 (1.8)	>.05

Abbreviation: CPR, cardiopulmonary resuscitation.

### Primary outcome: quality of CPR

Both groups demonstrated similar technical skills on the first training date. There was no statistically significant difference between the penalty points (Graham score) of the intervention group or control group, with mean 5.694 (95% confidence interval, CI [−16.821, 5.434]) penalty points lower for the control group *t*(110) = −1.014, *P* = .331.

After the first training, both groups improved their TS performance, reflected by the reduced penalty points. Like in the baseline training, there was no statistically significant difference between the penalty points of the intervention group or control group, with mean 3.939 (95% CI [−4.038, 11.917]) penalty points lower for the control group *t*(138) = 0.976, *P* = .331.

The intracohort analysis showed a significant improvement of TS of the control group *t*(49) = 2.229, *P* = .030, whereas the intervention group did not improve significantly *t*(59) = 1.794, *P* = .078.

The sub-scorings of the Graham score are depicted in Table 2. As shown, the intervention group mainly improved on the category “correct use and timing of defibrillation.” The control group, on the other hand, performed significantly better in the categories of “correct compression frequency,” “clear airway” as well as the sum score (overall performance).

**Table 2.** TS performance (pre and post) assessed with the Graham score.

GRAHAM SCORING			INTERVENTION GROUP (*N* = 60)						CONTROL GROUP (*N* = 50)					
	CRITERION	PENALTY POINTS	PRE-PENALTY POINTS	POST-PENALTY POINTS					PRE-PENALTY POINTS	POST-PENALTY POINTS				
			*M*(SD)	*M*(SD)	MD	95% CI		*P*	*M*(SD)	*M*(SD)	MD	95% CI		*P*
						LL	UL					LL	UL	
Category 1: checking for consciousness	Right/done	0	0.25(1.10)	0.00(0.00)	0.250	−0.034	0.534	.083	0.50(1.52)	0.30(1.20)	0.200	−0.294	0.694	.420
	Wrong/not done	5												
Category 2: inform others/call for help	Right/done	0	0.25(1.10)	0.00(.00)	0.250	−0.034	0.534	.083	0.10(0.71)	0.40(1.37)	−0.300	−0.746	0.146	.182
	Wrong/not done	5												
Category 3: Open Airway	Right	0	2.67(5.16)	2.33(5.64)	0.333	−1.738	2.405	.749	4.60(7.34)	1.00(4.17)	3.600	1.394	5.806	.002*
	Inadequate	10												
	No attempt/wrong	20												
Category 4: assess breathing	Right/done	0	4.92(7.51)	5.00(7.81)	−0.083	−3.225	3.058	.958	5.20(7.76)	3.80(6.43)	1.400	−1.556	4.356	.346
	Inadequate	5												
	Wrong/not done	20												
Category 5: circulation control	Right/done	0	5.17(7.76)	3.92(7.08)	1.250	−1.717	4.217	.403	8.00(9.58)	5.20(8.14)	2.800	−0.787	6.387	.123
	Inadequate	5												
	Wrong/not done	20												
Category 6: (CC) hand position	Right	0	2.00(4.80)	1.33(3.89)	0.667	−0.972	2.305	.419	2.80(5.36)	1.20(3.28)	1.600	−0.284	3.448	.088
	Wrong	10												
	Grossly wrong	20												
Category 7: (CC) frequency	100–120/min	0	1.25(2.18)	.58(1.62)	0.667	−0.065	1.398	.073	2.80(3.93)	0.60(1.64)	2.200	0.943	3.457	<.001*
	120–140/min or 80–100/min	5												
	>140/min or <80/min	15												
Category 8: (CC) depth	Right (5–6 cm)	0	3.33(5.10)	3.33(5.10)	0.000	−1.577	1.577	1.000	3.20(4.71)	3.60(5.63)	−0.400	−2.621	1.821	.719
	Too light (3–5 cm), too deep (>6 cm)	10												
	Wrong (<3 cm) or no compression	20												
Category 9: (CC) chest recoil	Right	0	2.58(3.38)	2.08(2.95)	0.500	−0.633	1.633	0.381	2.20(2.51)	1.50(2.90)	0.700	−0.208	1.608	.128
	Slightly incomplete (<2 cm)	5												
	Wrong (no recoil or >2 cm)	15												
Category 10: ventilation	Adequate (>400 ml)	0	6.00(7.18)	4.17(6.46)	1.833	−0.320	3.986	.094	3.80(6.02)	3.80(6.35)	0.000	−2.503	2.503	1.000
	Inadequate (<400 ml)	10												
	No or wrong (<200 ml)	20												
Category 11: use and timing of defibrillation	Right/used	0	3.33(7.51)	.67(3.62)	2.667	0.656	4.677	.010*	3.60(7.76)	1.60(5.48)	2.000	−0.631	4.631	.133
	Wrong/not used	20												
Category 12: drug administration (adrenaline)	Right timing	0	2.17(5.55)	1.83(4.32)	0.333	−1.507	2.173	.0718	2.20(4.19)	2.80(5.36)	−0.600	−2.539	1.339	.537
	Wrong timing	10												
	No preparation/not done	20												
		Mean score	33.92(26.73)	25.25(23.69)	8.667	−1.002	18.335	.078	39.00(32.92)	25.80(22.00)	13.200	1.302	25.098	.030*

*Note.* The pre-test scores are the baseline scores, assessed in the first training of both groups. The post-test scores were assessed in the second training (which was after the intervention in the intervention group). Abbreviations: TS, technical skills; SD, standard deviation; M, mean value; CI, confidence interval; LL, lower limit; UL, upper limit; MD, mean difference.

* The differences were significant at *P* < .005.

Both groups performed well to average on the three NTS dimensions. There was no statistically significant difference between both groups at the baseline training and after the second training (Table 3a). As further demonstrated in Table 3b, the intervention group enhanced their NTS significantly on Dimension 1, Dimension 2, and the sum scoring (overall performance), whereas the control group improved slightly on Dimension 2.

**Table 3a.** NTS performance (pre- and post-test) assessed with the AS-NTS (group comparison).

PRE-TEST									POST-TEST						
		INTERVENTION GROUP (*N* = 60), CONTROL GROUP (*N* = 50)									INTERVENTION GROUP (*N* = 60), CONTROL GROUP (*N* = 50)				
	*F*	*T*	DF	*P*	95% CI			MD	*F*	*T*	DF	*P*	95% CI		MD
					LL	UL							LL	UL	
D1	1.483	1.198	110	.234	−0.149	0.603		0.227	0.000	0.930	138	.354	−0.140	0.388	0.124
D2	0.221	0.401	110	.609	−0.295	0.445		0.075	0.037	0.518	138	.605	−0.195	0.333	0.069
D3	0.224	−0.101	110	.992	−0.374	0.370		−0.002	0.521	−0.507	138	.613	−0.346	0.205	−0.071

Abbreviations: NTS, non-technical skills; AS-NTS, Anaesthesiology Students’ Non-Technical Skills; CI, confidence interval; LL, lower limit; UL, upper limit; MD, mean difference.

**Table 3b.** NTS performance (pre- and post-test) assessed with the AS-NTS (intracohort analysis).

	INTERVENTION GROUP (*N* = 60)									CONTROL GROUP (*N* = 50)								
	PRE-TEST			POST-TEST			95% CI		*P*	PRE-TEST			POST-TEST			95% CI		*P*
	*M*	SD		*M*	SD	MD	LL	UL		*M*	SD		*M*	SD	MD	LL	UL	
D1	2.40	1.05		1.97	0.90	0.433	0.120	0.747	.008	2.16	0.96		1.96	0.70	0.20	−0.130	0.530	.229
D2	2.37	0.99		1.92	0.829	0.450	0.102	0.798	.012	2.28	0.99		1.92	0.75	0.360	0.012	0.708	.043
D3	2.27	1.02		1.92	0.89	0.350	−0.002	0.702	.051	2.26	0.970		2.04	0.78	0.220	−0.131	0.571	.213
S	7.03	2.84		5.80	2.32	1.23	0.296	2.17	.011	6.70	2.75		5.92	2	0.78	−0.169	1.73	.105

*Note.* The pre-test scores are the baseline scores, assessed in the first training of both groups. The post-test scores were assessed in the second training (which was after the intervention in the intervention group). Abbreviations: NTS, non-technical skills; AS-NTS, Anaesthesiology Students’ Non-Technical Skills; *M*, mean value; SD, standard deviation; D1, Dimension 1 of AS-NTS “planning tasks, prioritizing, and problem-solving”; D2, Dimension 2 of AS-NTS “teamwork and leadership”; D3, Dimension 3 of AS-NTS “team orientation”; CI, confidence interval; LL, lower limit; UL, upper limit; S, sum scoring of AS-NTS.

* The differences were significant at *P* < .005.

The correlation analysis between NTS and TS showed a strong positive correlation between NTS (sum score of the AS-NTS) and the Graham score, *r* = .599, *P *< .001, indicating that the higher (worse) the penalty points are the higher (worse) the NTS are.

### Secondary outcome

The motivational changes of both groups were comparable. Higher levels of intrinsic, identified, introjected, and autonomous motivation were reported by both groups at the post-training assessment, compared to their pre-training levels (Table 4).

**Table 4.** Situational motivation toward participating in the training.

	INTERVENTION GROUP (*N* = 69)								CONTROL GROUP (*N* = 56)								
	PRE-TEST		POST-TEST		*T*(DF)	95% CI		*P*		PRE-TEST		POST-TEST		*T*(DF)	95% CI		*P*
	*M*	SD	*M*	SD		LL	UL			*M*	SD	*M*	SD		LL	UL	
Intrinsic	4.591	1.140	4.866	1.150	−3.170(68)	−0.449	−0.102	.002		4.746	1.357	5.223	1.341	−5.380 (55)	−0.656	−0.300	<.001
Identified	5.522	1.037	5.783	0.878	−2.747 (68)	−0.450	−0.071	.008		5.482	1.039	5.885	0.966	−3.976 (55)	−0.606	−0.200	<.001
Introjected	3.498	1.197	3.779	1.426	−2.395 (68)	−0.516	−0.047	.019		3.875	1.378	4.232	1.522	−2.793 (55)	−0.613	−0.101	.007
Extrinsic	4.018	1.532	3.899	1.510	1.253 (68)	−0.701	0.310	.214		4.552	1.502	4.890	1.180	1.402 (55)	−0.075	0.427	.167
Amotivation	1.495	0.571	1.409	0.657	1.435 (68)	−0.033	0.205	.156		1.420	0.656	1.478	0.654	−0.679 (55)	−0.229	0.113	.500
Motivational indices																	
Autonomous	5.065	0.969	5.324	0.904	−3.485 (68)	−0.422	−0.115	<.001		5.114	1.119	5.554	1.076	−5.523 (55)	−0.600	−0.280	<.001
Controlled	3.758	1.208	3.839	1.235	−.976 (68)	−0.246	0.085	.333		4.214	1.225	4.304	1.225	−0.935 (55)	−0.285	0.104	.354

*Note.* The pre-test SIMS score was assessed prior to the first- and the post-test score prior to the second training (after the intervention for the intervention group). Abbreviations: M, mean value; SD, standard deviation; CI, confidence interval; LL, lower limit; UL, upper limit; SIMS, Situational Motivation Scale.

### Tertiary outcome

The control group and the intervention group reported significant learning gains on every scale of the CSA score.^44^ However the group comparison did not show a significant difference in the subjective learning gain between the two groups. Both groups started the first training session with similar pre-training scores and reported comparable subjective learning gains *t*(123) = −0.800, *P* = .425. The intracohort analysis for each dimension of the CSA is displayed in Table 5.

**Table 5.** Subjective learning gain reported by undergraduates.

ITEMS		INTERVENTION GROUP	MEAN LEARNING GAIN POINTS	*P*
		CONTROL GROUP		
1	I feel confident to detect and treat respiratory emergencies	Intervention (*n* = 73)	0.896	<.001
		Control (*n* = 56)	0.926	<.001
2	I feel confident to describe the correct sequence of treatments of the ACLS and BLS algorithms	Intervention (*n* = 75)	0.638	<.001
		Control (*n* = 58)	0.786	<.001
3	I feel confident to use the ACLS and BLS algorithm	Intervention (*n* = 75)	0.710	<.001
		Control (*n* = 58)	0.857	<.001
4	I feel confident to detect an irregular breathing	Intervention (*n* = 74)	0.279	.012
		Control (*n* = 58)	0.464	<.001
5	I feel confident to detect a cardiac arrest	Intervention (*n* = 75)	0.290	.007
		Control (*n* = 58)	0.429	<.001
6	I feel confident to clear the patient's airway	Intervention (*n* = 75)	0.420	.002
		Control (*n* = 57)	0.418	<.001
7	I feel confident to provide mask ventilation	Intervention (*n* = 75)	0.449	<.001
		Control (*n* = 58)	0.286	.020
8	I feel confident to perform high-quality chest compressions	Intervention (*n* = 75)	0.188	.047
		Control (*n* = 58)	0.375	<.001
9	I feel confident with the use of the defibrillator	Intervention (*n* = 75)	0.275	.012
		Control (*n* = 58)	0.250	.051
10	I feel confident being able to provide ACLS when I find an unresponsive patient	Intervention (*n* = 75)	0.738	<.001
		Control (*n* = 58)	0.607	<.001
11	I feel able to identify reversible causes of resuscitation	Intervention (*n* = 73)	0.403	<.001
		Control (*n* = 58)	0.625	<.001
12	I feel able to name life-threating circulatory disorders	Intervention (*n* = 75)	0.681	<.001
		Control (*n* = 58)	0.625	<.001
13	I feel able to lead ACLS in a team	Intervention (*n* = 75)	0.942	<.001
		Control (*n* = 56)	0.103	<.001
14	I feel able to pay attention to psychosocial skills in emergency situations and implementing them effectively	Intervention (*n* = 75)	0.435	<.001
		Control (*n* = 58)	0.661	<.001

Abbreviations: BLS, Basic Life Support; ACLS, Advanced Cardiac Life Support.

## Discussion

Our prospective randomized controlled study showed that SMM positively influence the development of NTS during ACLS training. However, the study also highlights the importance of hands-on practice for improving TS: The intervention group improved their NTS significantly, whereas the control group slightly improved on one skill dimension. Significant progress of TS was only observed within the control group.

A possible explanation for the TS improvement of the control group may lie in the greater hands-on time, which was due to our study design. As the time limit for our classes was 120 min per training and the intervention took at least 30 min, the control group had more time for haptic training and feedback. Our assumption is supported and backed up by emphasizing the dimensions of the TS (scoring) on which an improvement has been achieved within the control group. These TS, “correct compression frequency,” “correct hand position,” and “clear airway,” are developed purely through tactile experience, and can therefore be acquired with frequent practice or, in our context, through extended hands-on time.^45^ Purely haptic skills are enhanced by solely repetition, especially when it comes to non-complex practical skills, motor activity and the coordination of the motor cortex are enhanced through repetition.^46^ This explanation is further reinforced by fundamental principles of learning psychology: According to Milleŕs pyramid model^47^ and constructive alignment,^48^ clinical competencies can be divided into four hierarchical processes. Therefore, teaching and testing different levels of competencies should be adapted to the corresponding skill level.

Performing effective TS during resuscitation involves mastering the first three levels as described by Miller: 1. Knowledge, 2. application of knowledge, and 3. clinical skills competency.^47^ The learning cycle of these skills is accelerated by practice, repetition, and diligence,^47^ similar to the increased hands-on time of the control group.

In contrast to TS, acquiring NTS—which represents the final level of Miller's pyramid (“clinical performance”)—requires the transfer of knowledge into behavior.^49^ Since NTS are separate from medical knowledge and do not necessarily improve with experience and repetition,^50^ other approaches such as deep learning, reflection, and the creation of MM are essential for accelerating the development of NTS.^49^

Our intervention aimed to create SMM among team members, thereby fostering reflection and deep learning by sharing internal representations of real patterns, such as team tasks, responsibilities, and task distribution.^17,18^

Targeting NTS during CPR training is essential, as NTS have been identified as one of the five key focus areas in healthcare simulation to improve patient safety.^51^ The European Resuscitation Council Guidelines for Resuscitation explicitly recommend integrating NTS into ACLS training.^15^ However, there is a lack of evidence on how to effectively convey NTS.^5,6^ What has been demonstrated is the superiority of simulation-based medical education (SBME) over traditional didactic methods like seminars, making SBME a recognized approach for teaching resuscitation skills.^52,53^ Therefore, advanced didactic methods should be identified and incorporated into SBME to enhance the dissemination of NTS.^54,55^ Based on our results, SMM into simulation teaching may be one such effective approach.

Interestingly, the intervention group improved significantly on the sum score, Dimensions 1 and 2 (“Planning tasks, prioritizing and problem-solving”; “Teamwork and leadership”) and no relevant improvement on the third dimension of AS-NTS (“Team orientation”) was observed.^56^

The reason why the students improved in teamwork and leadership but not in team orientation can be explained from different perspectives. Leadership can be divided into two main skill components, which are distinguished by AS-NTS, many other taxonomies, and behavioral leadership models, such as the leadership grid. The two main components of leadership are considered separately:^56^ “team orientation” involves coordinating and motivating team members, while “task orientation” focuses on managing the workload.^57,58^ In this context, “team orientation” (Dimension 3 of AS-NTS) emphasizes the processes of building a team, whereas “teamwork and leadership” (Dimension 2 of AS-NTS) highlights the processes involved in completing a task.^56^

Teamwork skills are expressed based on behavioral precursors and attributes.^59,60^

In our study, key precursors include “information sharing” and “understanding professional roles,” with information sharing represented by the first dimension of AS-NTS (“Planning tasks, prioritizing, and problem-solving”).^60^ Our intervention primarily targeted communication and task coordination during emergencies, leading to significant improvements in task-related teamwork skills. The creation of SMM enhanced decision-making, a crucial teamwork attribute.^18^ The intervention appeared to specifically enhance leadership skills related to task performance (Dimension 2 of AS-NTS), which, as shown in a previous study, do not solely improve through repetition, as evidenced by minimal skill enhancement in “teamwork and leadership” among students in emergency simulation training with a spiral curriculum.^61,62^

Both groups experienced an increase in autonomous motivation after the first training, with no significant differences between them. This highlights the effectiveness of SBME in engaging medical students with the task.^24^ Notably, the intervention group also reported a significant rise in introjected motivation.^24^

Although introjected motivation is more autonomous than external regulation, it still belongs to the controlled motivational circle, and therefore, the corresponding activity or task is seen as accustomed by others.^30,31^ Introjected behavioral regulation is predominant when attaining self-esteem and citing ego.^29,31,32^ The mental modeling approach was unfamiliar to the medical students, introducing them to new learning content. This novelty may have led to a sense of helplessness, during which strategies to maintain self-esteem became more prominent, resulting in increased introjected motivation. However, although autonomous motivation is aspired in medical education,^24^ our study rules out the negative effects of controlled motivation and conversely of our intervention. Studies indicate that autonomous and introjected regulation can coexist and still positively impact job characteristics and well-being. Similar findings from sports suggest that motivation across the continuum enhances performance. This was also seen in job perfectionism and workaholism, where broader motivation led to higher trait levels.^63,64–65^

The efficacy of our training in general was also reflected by the reported subjective learning gains of the undergraduates. Although self-perception forms the basis, learning gain can be regarded as an indirect indicator of actual skill enhancement, as strong correlations between subjective learning gain (CSA) and objective learning gain, measured through summative assessments, have been reported.^44^

Both groups reported significant learning gains for every dimension of the CSA and there were no differences between the groups. This indicates that the adapted CSA might not have been sensitive enough to discriminate the learning gains between the groups regarding NTS. Herein lies a limitation of our study. Only two CSA dimensions targeted NTS, and the questions were very constrained and not specific enough and only addressed general NTS that improve during ACLS training without considering more advanced NTS that were conveyed by the intervention (“I feel able to lead ACLS in a team”; “I feel able to pay attention to psychosocial skills in emergency situations and implementing them effectively”). An expanded pilot phase of the CSA might have led to further adaptations and better discrimination between the groups. Therefore, subjective learning gain should be considered an indirect surrogate assessment for actual learning gain, and actual performance (TS and NTS) should be measured with adapted and specific tools.

One might argue that the assessment of TS using an observational method (Graham scoring) rather than a real-time feedback device has resulted in less representative data. The best approach would have been to integrate the strengths of both human judgment (assessment) and technology (CPR meters). Although research has demonstrated that real-time feedback enhances CPR performance assessment,^66^ it has also been highlighted that there is still a need for human observational abilities for aspects that devices are unable to quantify, including awareness, breathing, circulation control, and the overall algorithm.^67^ Furthermore, given that some manufacturers highlight the error-proneness of CPR meters, their abandonment in favor of human assessment using a validated instrument (the Graham score) was considered an acceptable approach.^41^

Further limitations of our study merit consideration. The primary goal of CPR is to minimize the “no-flow time”—the period during which no blood flow occurs—and to ensure the prompt use of an automated external defibrillator (AED).^4,68–70^ The Graham score, however, only evaluates AED use and does not address the time between CPR measures.^41^ Although the Graham scoring system covers important aspects of CPR, it fails to adequately incorporate the critical performance marker of ”no-flow time,” which is essential for improving neurological outcomes in patients.

For assessing the training effects on patient outcomes, further CPR performance markers need to be evaluated and expanded scoring systems developed.

It is considered highly unlikely that the results of the studies would have been affected by a possible Hawthorne effect. Firstly, the students were unaware of which study arm they had been allocated due to the blinding process. Secondly, our faculty engages in a significant amount of teaching research in the field of emergency medicine, which means that the undergraduates were not exposed to a novel situation in the present study. Additionally, it is challenging to regulate onés behavior in stressful situations such as CPR.^71^ Although our study was conducted in a single-center design, we believe that our study results have a certain generalizability regarding the effectiveness of SMM during (undergraduate) ALS training. Nevertheless, for a broader generalizability, further studies need to confirm our results while considering different independent variables like the experience of the instructors and undergraduates, as well as the curriculum itself. Also, follow-up studies need to explore the long-term effect of SMM, as it is already known, that skill deterioration after CPR training starts within a few weeks.^6,72^

In summary, the approach of mental modeling, as an integrated part of SBME, seems to enhance and accelerate the transformation of behavioral benchmarks into one’s behavioral patterns, which in turn equates the acquisition of NTS.^73^ Although many other studies have targeted enhancing NTS, an effective approach has not yet been identified.^45,74^ The reason why NTS could not be conveyed sufficiently by the described interventions can be explained by learning psychology. As many studies focused on post-simulation debriefings, the participating undergraduates might not have had the emotional absorption capacity for the debriefing inputs. This in turn leads to high cognitive load which hampers the learning cycle.^75,76^ To foster learning from experience, learning concepts need to be considered beyond the boundary of formal instructional designs, as the SBME itself is an isolated instructional design and might not provide the ideal learning setting. Learning needs flexibility and does not occur by the instructor providing information and the undergraduate simply absorbs it.^76,77^ The mental modeling approach considers different facets of learning and targets ideally learning which is detached from the factual approach.^19,21,22^

There was a significant correlation between team performance (NTS) and technical skills. Herein lies an important implication of our findings: NTS are important skills that complement TS to ensure patient safety,^78^ and therefore, they should be conveyed and targeted equally as an integral part of resuscitation training.^5^

## Conclusion and implications

SBME is effective for teaching TS, but integrating advanced didactic methods is necessary to better address and complement NTS. The creation of SMM during emergency training is one approach that improves NTS, but hands-on practice should not be limited. Faculty development programs, such as “train-the-trainer” initiatives, should incorporate dedicated courses to prepare ACLS instructors for the teaching intervention of structured SMM during simulation-based training. The curriculum design should include at least two ACLS training sessions to ensure optimal learning outcomes. The initial training session should focus on traditional instruction, allowing sufficient hands-on time for undergraduates to become familiar with the manikins and the simulation environment. A subsequent session could serve as a refresher, reinforcing practical skills while emphasizing the development of NTS through the integration of SMM interventions. ACLS training should target to convey TS and NTS equally, as they are correlated and enhance the quality of CPR.

## Supplemental Material

sj-doc-1-mde-10.1177_23821205251316749 - Supplemental material for The Creation of Shared Mental Models in Simulation Training Enhances Quality of Resuscitation: A Randomized Controlled Study Supplemental material, sj-doc-1-mde-10.1177_23821205251316749 for The Creation of Shared Mental Models in Simulation Training Enhances Quality of Resuscitation: A Randomized Controlled Study by Christopher Friederich, Leonie Schulte-Unetrop, Denisa Cenaj, Leonie Fée Kröger, Josephine Küllmei, Christian Zöllner and Parisa Moll-Khosrawi in Journal of Medical Education and Curricular Development
